# Analysis of potential TAK1/Map3k7 phosphorylation targets in hypertrophy and cachexia models of skeletal muscle

**DOI:** 10.1242/bio.060487

**Published:** 2024-09-25

**Authors:** Fatemeh Nasehi, Cameron Rylance, Erin Schnell, Maslyn Ann Greene, Caroline Conway, Zachary Hough, Susan Duckett, Robin C. Muise-Helmericks, Ann Catherine Foley

**Affiliations:** ^1^ Department of Bioengineering, Clemson University, 68 President Street, Charleston, SC 29425, USA; ^2^ University of South Carolina School of Medicine, 6311 Garners Ferry Road, Columbia, SC 29209, USA; ^3^ Department of Animal and Veterinary Science, Clemson University, Lane #129, Clemson, SC 29634, USA; ^4^ Dartmouth's Department of Cognitive Science, 5 Maynard St, Hanover, NH 03755, USA; ^5^ University of Maryland, Baltimore School of Medicine, Baltimore, MD 21201, USA; ^6^ Department of Regenerative Medicine and Cell Biology, Medical University of South Carolina, 173 Ashley Avenue, Charleston, SC 29425, USA

**Keywords:** TGFβ-activated kinase (TAK1), Kinase signaling, Texel sheep, Muscle hypertrophy, Cachexia

## Abstract

TGFβ-activated kinase-1 (TAK1) is phosphorylated during both muscle growth and muscle wasting. To understand how this can lead to such opposite effects, we first performed multiplex kinase array of mouse embryonic stem cells with and without stimulation of TAK1 to determine its potential downstream targets. The phosphorylation of these targets was then compared in three different models: hypertrophic longissimus muscle of Texel sheep, tibialis anterior muscle of mice with cancer-induced cachexia and C2C12-derived myofibers, with and without blockade of TAK1 phosphorylation. In both Texel sheep and in cancer-induced cachexia, phosphorylation of both TAK1 and p38 was increased. Whereas p90RSK was increased in Texel sheep but not cachexia and the phosphorylation of HSP27 and total Jnk were increased in cachexia but not Texel. To understand this further, we examined the expression of these proteins in C2C12 cells as they differentiated into myotubes, with and without blockade of TAK1 phosphorylation. In C2C12 cells, decreased phosphorylation of TAK1 leads to reduced phosphorylation of p38, JNK, and HSP27 after 16 h and muscle fiber hypertrophy after 3 days. However, continuous blockade of this pathway leads to muscle fiber failure, suggesting that the timing of TAK1 activation controls the expression of context-dependent targets.

## Teaser

**Summary:** Identification of TAK1 downstream targets essential for muscle growth and hypertrophy has broad implications for understanding muscle diseases including the identification of potentially druggable targets for those diseases.

## INTRODUCTION

Dysregulation of TGFβ signaling downstream of myostatin, mainly through the TGF-β-activated kinase 1 (TAK1), is believed to be pivotal in muscle hypertrophy (Hindi et al., 2018). Conversely, TAK1 activation is also involved in muscle-wasting diseases (Srivastava et al., 2007; Roy et al., 2023). Understanding the pathways downstream of TAK1 that regulate these different outcomes will be essential to understanding diseases that impact muscle size (Carnac et al., 2007).

Muscle overgrowth can involve both over-proliferation (hyperplasia) and increased size of muscle fibers (hypertrophy). Muscle overgrowth is beneficial in the context of primary food animals (Talebi et al., 2022) such as Texel sheep, a domestic breed marked by significant hypertrophic growth of skeletal muscle (Liu et al., 2012). Muscle hypertrophy in Texel sheep is thought to be due to a single point mutation in the myostatin gene, creating a novel binding site for the microRNAs miR-1 and miR-206 (Clop et al., 2006). Myostatin is a Transforming Growth Factor β (TGFβ) family member that is a negative regulator of skeletal muscle growth (Biesemann et al., 2015). Phosphorylation of TAK1 is thought to mediate this phenotype (Chen et al., 2021), but this has not been shown directly in Texel sheep (Hindi et al., 2018).

Cachexia is a muscle-wasting disease characterized by skeletal muscle loss. It is a common side effect of cancer. Understanding the etiology of this disease could provide relief to cancer patients; however, the mechanisms underlying this disease are poorly understood due to a lack of appropriate animal models. For these studies, we use the established pancreatic ductal adenocarcinoma (KPP) model of cancer cachexia. This model is superior to other mouse models due to its genetic and tissue-level similarity to human cachexia patients (Talbert et al., 2019). It is assumed that TAK1 signaling will play an important role in this model, but this has yet to be shown. Also, it should be noted that signaling differences seen in these samples could be due to inflammation or other processes that result from cachexia.

Here, we identified phosphorylation targets of TAK1 by kinase array and studied their expression and phosphorylation status in models of muscle hypertrophy and muscle cachexia to better understand the role of TAK1 and its downstream targets in these processes. These kinases were also examined during myocyte differentiation in C2C12 cells (Yaffe and Saxel, 1977). These studies confirm that phosphorylation of TAK1 and p38 plays roles in muscle hypertrophy and cachexia. Studies in C2C12 cells suggest phospho-TAK1 plays distinct roles at different stages of muscle fiber differentiation and growth.

## RESULTS

### Phospho-array response to TAK1 overexpression

**Fig. 1.**
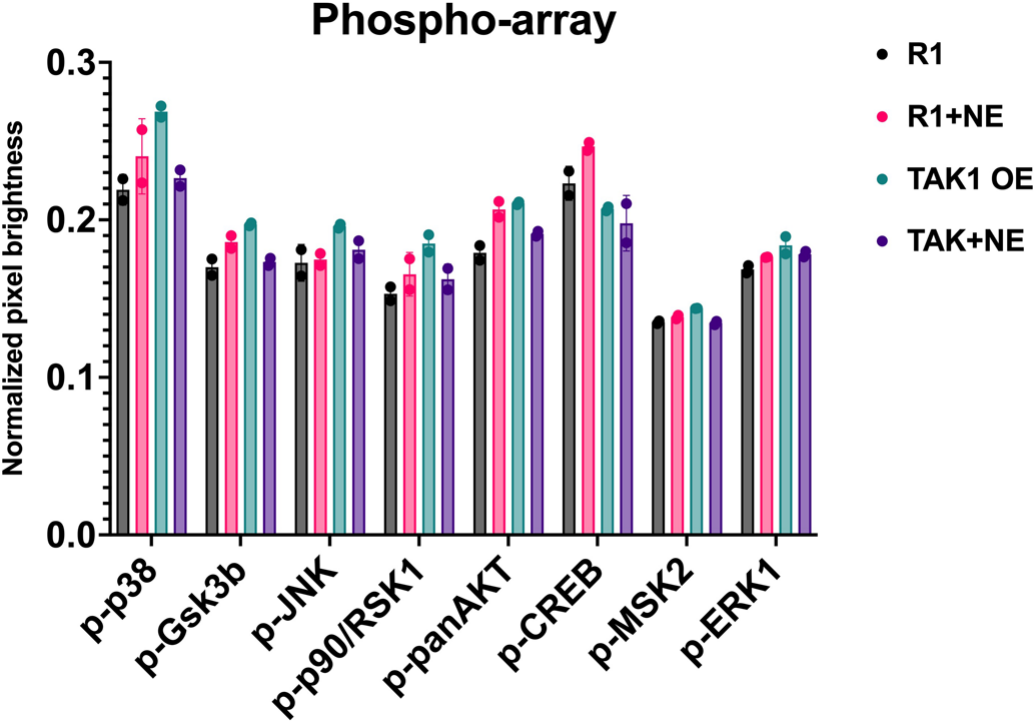
**Analysis of TAK1 phosphorylation and its impact on downstream targets using RayBio C1-Series human and mouse MAPK pathway phosphorylation array.** Wild-type (WT) mESC (mouse embryonic stem cells), WT mESC +NE (mouse embryonic stem cells that are stimulated with norepinephrine), TAK1OE (Map3k7/TAK1-overexpressing mouse embryonic stem cells). TAK1OE+ NE (Map3k7/TAK1-overexpressing mouse embryonic stem cells stimulated with norepinephrine). Error bars represent standard deviation from two technical replicates.

To specifically identify kinases that are downstream targets of TAK1, we examined the phosphorylation status of MAP kinases (p38, GSK3B, JNK, p90RSK1, p90RSK2, panAkt, CREB, Msk2, and Erk1) in wild-type embryonic stem (ES) cells (WT mESC), TAK1-overexpressing ES cells (TAK1OE), WT ES cells treated with norepinephrine (WT mESC+ NE), and TAK1-overexpressing ES cells further stimulated with NE (TAK1OE+ NE) (Fig. 1) using the RayBiotech C1-Series Human and Mouse MAPK pathway phosphorylation array. All kinases with changes in the phosphorylation status greater than the standard deviation of the trial are shown. In addition, a few with no significant change are included in Fig. 1. Stimulation of the TAK1 signaling pathway, either by TAK1 overexpression or treatment with norepinephrine, increased the phosphorylation of several MAP kinases, including well-described targets such as p38 and some novel targets such as HSP27, p90RSK, and CREB. However, our results showed that when TAK1-overexpressing mESCs were also treated with norepinephrine, the phosphorylation of most downstream targets decreased. This suggests that supraphysiological activation of TAK1 may cause the downregulation of its downstream targets via negative feedback loops.

### TAK1 signaling targets in skeletal muscle of Texel sheep

**Fig. 2.**
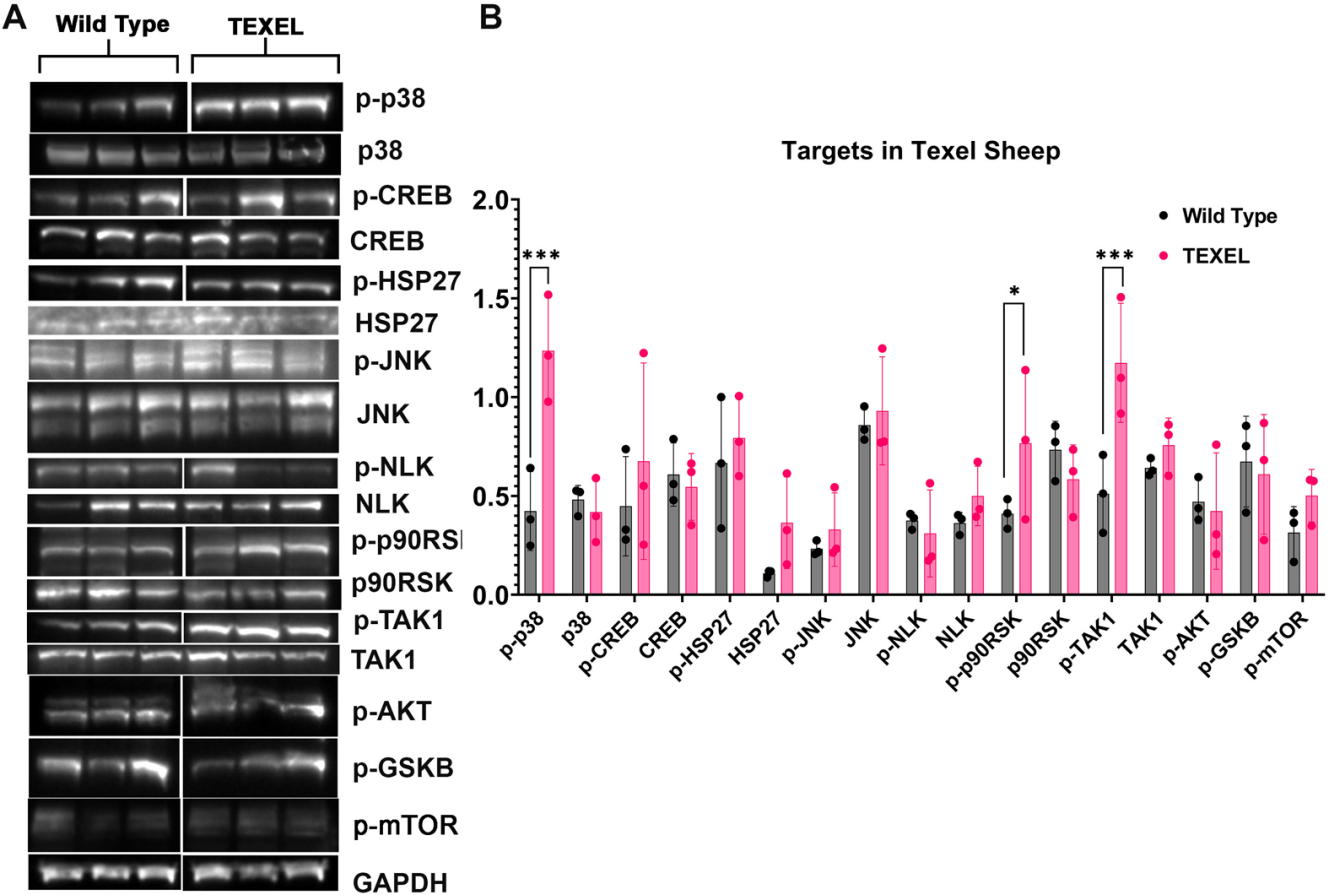
**TAK1 signaling pathways in Texel sheep muscle.** (A) Western blots of p-p38, p38, p-CREB, CREB, p-HSP27, HSP27, p-JNK, and JNK, p-NLK, NLK, p-p90RSK, p90RSK, p-TAK1, TAK1, p-AKT, p-GSK3B, and p-mTOR in WT and Texel sheep. White lines mark where an empty lane was removed from the images of some blots. (B) Quantitative analysis of p-p38, p38, p-CREB, CREB, p-HSP27, HSP27, p-JNK, and JNK, p-NLK, NLK, p-p90RSK, p90RSK, p-TAK1, TAK1, p-AKT, p-GSK3B, and p-mTOR in WT and Texel sheep. (Pixel density normalized to GAPDH). Texel sheep are GS1L1 LM, GS8 LM, G19L1 LM. ***P<0.001, *P<0.05.

Since signaling pathway regulation is highly context-dependent, we tested putative TAK1 phosphorylation targets *in vivo*. Muscle hypertrophy in Texel sheep is linked to a point mutation in myostatin and works by misregulation of TAK1 and other downstream effectors, including p38, JNK1, and JNK2 (Chen et al., 2021; Biesemann et al., 2015). To assess TAK1 phosphorylation targets in Texel sheep longissimus muscle, samples were collected from both Suffolk/WT (*n*=3) and Texel sheep (*n*=3) and putative targets from our microarray analyzed by western blotting (Fig. 2A,B) for both total protein expression and expression of their phosphorylated forms. Expression of p-CREB, p-HSP27, HSP27, p-JNK, JNK, and p-mTOR proteins was not statistically different in the longissimus muscle of Texel sheep compared to Suffolk sheep. However, there was a significant increase in the expression of p-p38, p-TAK1, and p-p90RSK, even though there was no statistically significant change in the expression of total protein for p38, TAK1, and p90RSK (Fig. 2A,B). These findings confirm the dysregulation of TAK1 phosphorylation in Texel sheep. The upregulation of p-p38 and p-p90RSK in these sheep implicates these kinases as potential mediators of muscle hypertrophy.

### TAK1 signaling targets in skeletal muscle of cancer cachexia mice

**Fig. 3.**
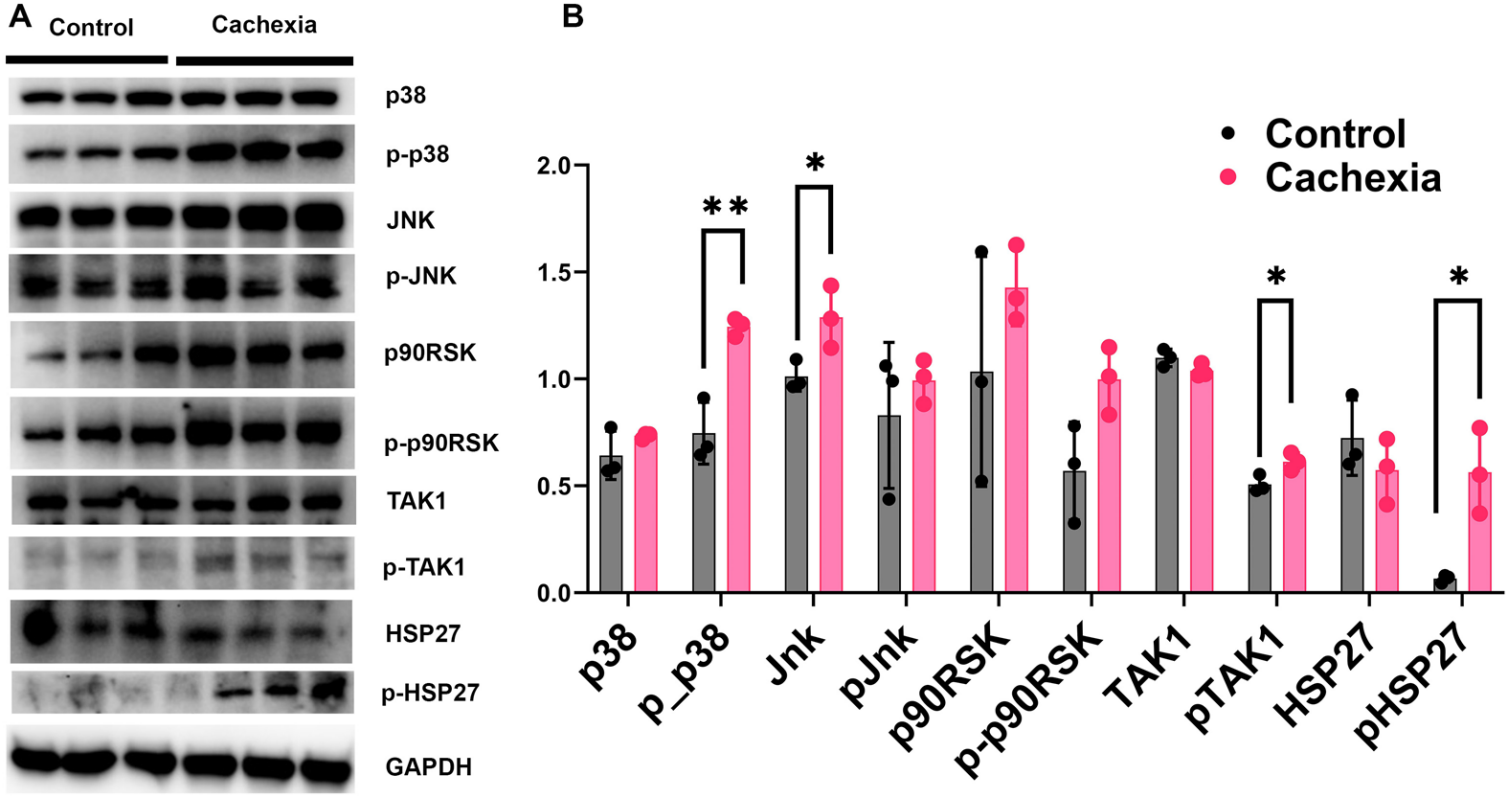
**Western blot analysis of Cachexia samples.** (A) Western blot of p38, p-p38, JNK, p-JNK, p90RSK, p-p90RSK, TAK1, p-TAK1, HSP27, p-hsp27. (B) Quantitative analysis of p38, p-p38, JNK, p-JNK, p90RSK, p-p90RSK, TAK1, p-TAK1, HSP27, and p-HSP27. (Pixel density normalized to GAPDH). **P<0.01, *P<0.05.

Tibialis anterior muscle was excised from cancer cachectic and control mice (*n*=3) (Talbert et al., 2019). Western blots were performed using antibodies against potential targets of TAK1 (Fig. 3). We found significant increases in the phosphorylation of TAK1 and p38 without changes to the total amount of these proteins. In addition, we saw increases in phosphorylation of HSP27 as well as an increase in total JNK. These data show a link between activated TAK1 and p38 in this model. We also identify HSP27 and JNK as potential context-dependent targets.

### TAK1 signaling targets in differentiating C2C12 cells

**Fig. 4.**
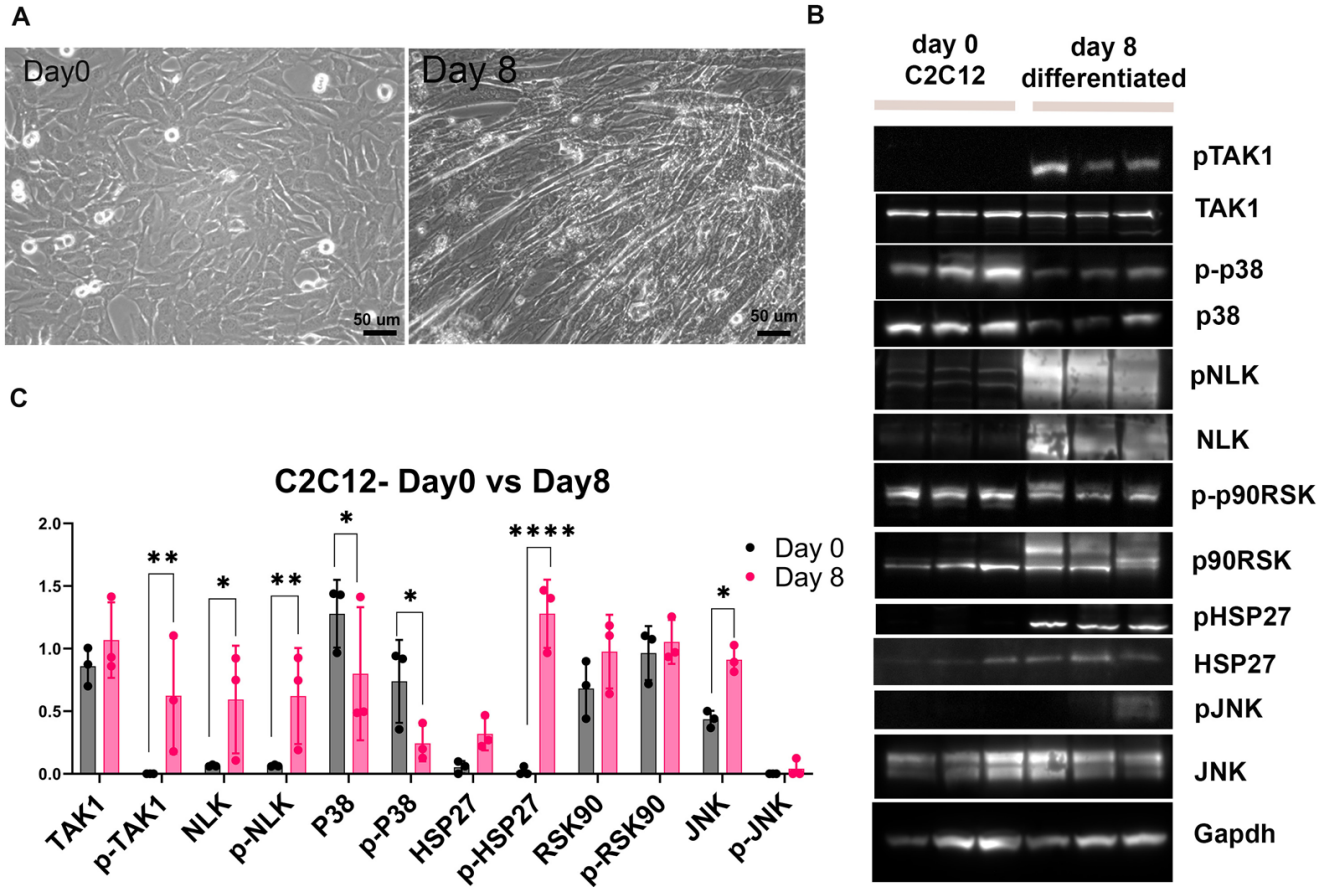
**TAK1 signaling pathways in differentiated C2C12 cells.** (A) Myotube formation after plating C2C12 cells at day 0 and day 8. (B) Western blots of p-TAK1, TAK1, p-p38, p38, p-NLK, NLK, p-p90RSK, p90RSK, p-HSP27, HSP27, p-JNK, and JNK in C2C12 cells at day 0 and day 8. (C) Quantitative analysis of TAK1, p-TAK1, NLK, p-NLK, p38, p-p38, HSP27, p-HSP27, p90RSK, p-p90RSK, JNK, and p-JNK2. (Pixel density normalized to GAPDH). Scale bars: 50 μm. ****P<0.0001, ***P<0.001, **P<0.01, *P<0.05 significance.

To further explore how phosphorylation of TAK1 might result in both muscle hypertrophy and muscle wasting, we investigated it and its potential downstream targets in C2C12 cells during muscle differentiation. As previously reported, C2C12 cells start as single cells but fuse and form myotubes within 8 days of differentiation (Fig. 4A) (Yaffe and Saxel, 1977). Cells were collected at day 0 and day 8 of differentiation to evaluate how the expression of TAK1 targets changes during this process (*n*=3) (Fig. 4B). p-TAK1, NLK, p-NLK, p-HSP27, and JNK were significantly upregulated in differentiated C2C12 samples compared to undifferentiated samples (day 0) (Fig. 4B,C). However, the expression of p38 and p-p38 were downregulated in differentiated C2C12 cells. This suggests that p38 is involved in processes that dysregulate muscle fibers but are actively repressed during normal muscle differentiation.

### Treatment of C2C12 cells with the TAK1 inhibitor, 5z-7-oxozeanol (OXO), increased the diameter of myotubes in culture and affected overall muscle growth

**Fig. 5.**
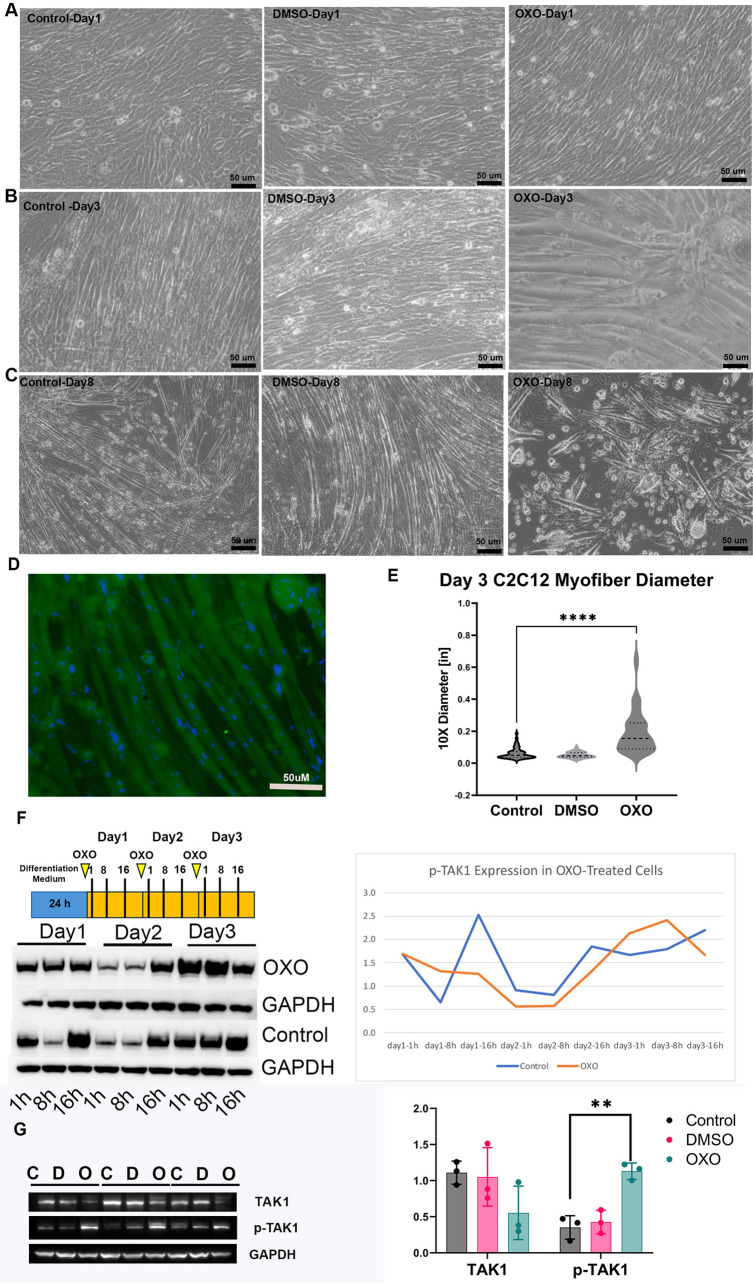
**Differentiated C2C12 cells treated with DMSO and OXO.** Differentiated C2C12 cells were treated by insulin (control), DMSO, and OXO dissolved in DMSO after 1 day (A), 3 days (B), and 8 days (C). Scale bars: 50 μm. (D) Immuno-cytochemistry for MYF5 at day 3 to confirm myofiber differentiation. Scale bar: 50 μm. (E) Quantitative analysis of muscle size due to treatments. (F) Scheme of treatment regime with OXO treatments and collection times indicated, and western blots of p-TAK1 when C2C12 cells were treated with OXO within 3 days. The line graph indicates the quantification of a single time course with control indicated in blue and OXO-treated orange. (G) Western blots of TAK1 and p-TAK1 with or without OXO at day 8, with quantification. (Pixel density normalized to GAPDH). ****P<0.0001, **P<0.01 significance.

To determine the role of TAK1 phosphorylation in myotube formation, we repeated these experiments using OXO to block TAK1 phosphorylation. C2C12 cells were differentiated as described above. In parallel, cells were treated with either 100 nM OXO or carrier control (DMSO) for 8 days. In our initial experiments, we observed that differentiation in the presence of OXO completely inhibited muscle fiber differentiation, suggesting that the initial differentiation of myotubes requires TAK1-phosphorylation. When OXO was added starting 2 days into the differentiation protocol, myotube formation proceeded normally. For all remaining studies, OXO was added a day, with fresh OXO added daily. Cells were observed and collected on days one, three, and eight after treatment to examine TAK1's effect on myofiber formation. On day one after treatment, OXO-treated samples were identical to controls (Fig. 5A). By day 3, OXO-treated myofibers were significantly larger in diameter (Fig. 5B). By day 8, muscle fibers that had been continuously treated with OXO (fresh OXO added daily) had broken down and separated into single cells (Fig. 5C). Myofiber differentiation at day three was confirmed by MYF5 immunocytochemistry (Fig. 5D). Myofiber diameter was quantified under each condition using ImageJ, confirming that OXO treatment increased myofiber diameter on day 3 (Fig. 5E). This is inconsistent with data showing increased TAK1 phosphorylation in the hypertrophic muscle of Texel sheep.

We have shown previously that TAK1 can self-regulate its activation by initiating a downstream feedback loop (Brown et al., 2017; Dai et al., 2023). We hypothesized that OXO treatment in this model of differentiation may be doing the same thing. To confirm this, we examined TAK1 phosphorylation in differentiating C2C12 cells with or without OXO until day 3. TAK1 phosphorylation did decrease within 18 h relative to controls, but by day 3, continuous OXO treatment caused an increase in phospho-TAK1 at the same point at which we observed muscle fiber hypertrophy (Fig. 5F). Consistent treatment of these samples with OXO to day 8 resulted in no change to total TAK1 but an increase in phospho-TAK1 (*n*=3) (Fig. 5G). These data are consistent with our previous data showing that TAK1 can self-regulate its activation. These data also explain why OXO treatment resulted in increased muscle fiber diameter at day 3. Briefly, these data suggest that increased TAK1 phosphorylation is highly correlated with increased muscle fiber diameter but that long-term exposure to situations with increased activation of TAK1 decreases muscle fiber integrity.

### Blockade of TAK1 caused downregulation of p-p38, p-JNK, and p-HSP27

**Fig. 6.**
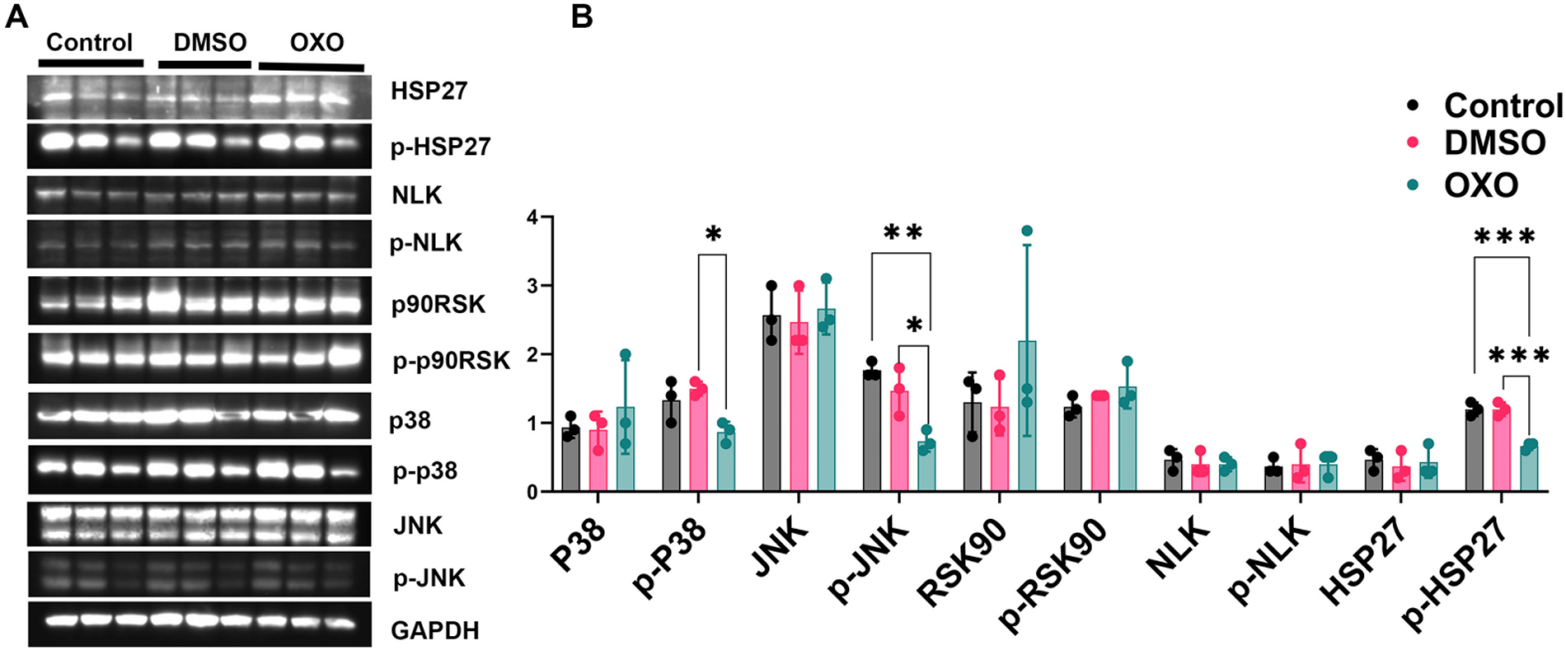
**Western blot analysis of C2C12 cells on 16** **h after OXO treatment.** (A) Western blots of HSP27, p-HSP27, NLK, p-NLK, p90RSK, p-p90RSK, p38, p-p38, and JNK, p-JNK. (B) Quantitative analysis of p38, p-p38, JNK, p-JNK, p90RSK, p-p90RSK, NLK, p-NLK, HSP27, and p-HSP27. (Pixel density normalized to GAPDH). ***P<0.001, **P<0.01, *P<0.05.

Since our data indicate that OXO's ability to block TAK1 phosphorylation cannot be maintained consistently in this model of cellular differentiation, we tested the phosphorylation status of downstream targets at 16 h after treatment. At this point, the OXO blockade of TAK1 is maximal. Cells were collected from both OXO-treated and control cultures and assessed for changes in the phosphorylation of MAP kinase proteins (*n*=3) (Fig. 6). We observed that p-p38, p-JNK, and p-HSP27 were downregulated when TAK1 phosphorylation is decreased (two-tailed *t*-test; p-p38 *P*=0.02, p-JNK *P*=0.004, p-HSP27 *P*=0.002) (Fig. 6A,B). There were no significant changes in other downstream targets of TAK1, such as p90RSK and p-p90RSK. There were no significant changes in carrier control samples (Fig. 6B).

These data support the hypothesis that the phosphorylation of p38, Jnk and Hsp27 depends on the phosphorylation status of TAK1 and, therefore, are direct or indirect targets.

## DISCUSSION

**Fig. 7.**
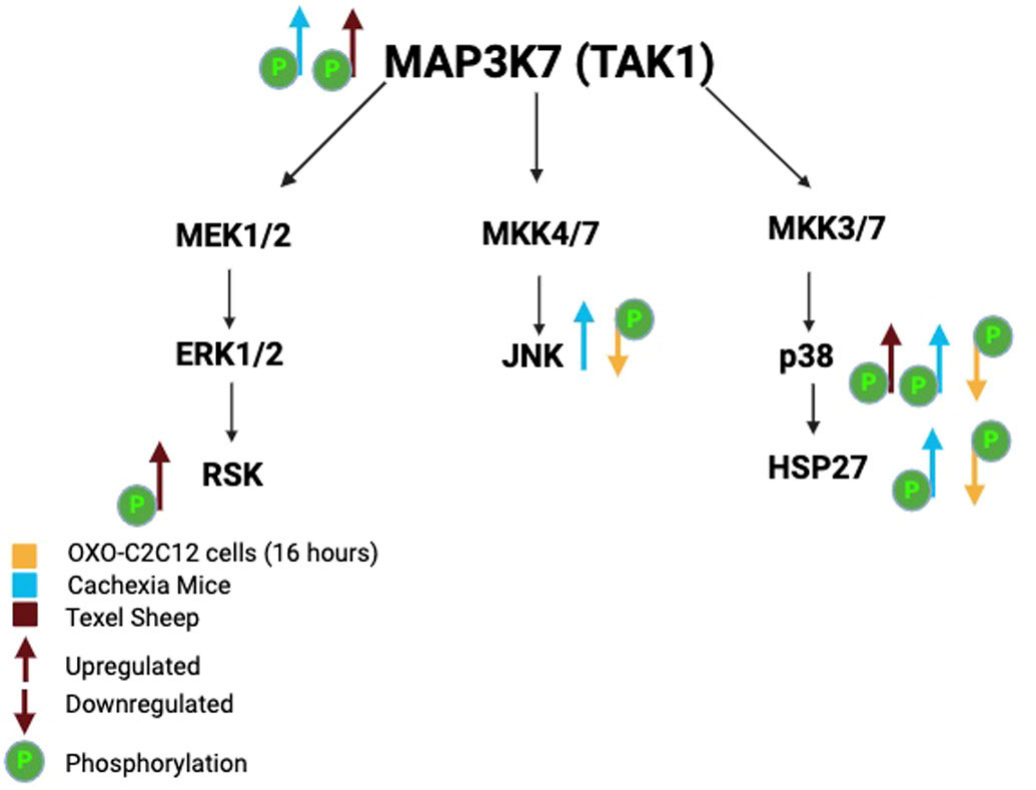
**Expression of different downstream TAK1 targets**. Different downstream TAK1 targets in Texel sheep, cachexia mice, and C2C12 cells treated with OXO 16 h after treatment.

TAK1 is a member of the MAP kinase family that mediates context-dependent activation of distinct signaling pathways (Hindi et al., 2018). TAK1 is downstream of myostatin in muscle growth and hypertrophy, negatively regulating skeletal muscle growth (Carnac et al., 2007). Interestingly, in the longissimus muscle of adult Texel sheep, there was a significant increase in the phosphorylation of TAK1 and other kinases that are TAK1 targets, including p38 and p90RSK (Fig. 7). This, at first, seems surprising given that myostatin has been shown to regulate TAK-1 phosphorylation (Chen et al., 2021). However, our previous research consistently demonstrated that regulating targets downstream of TAK1 is extraordinarily complex. Previously, we showed that forced overexpression of TAK1 suppresses the transcription of endogenous TAK1 (Brown et al., 2017). In support of this idea, Hunter et al. documented that chronic overexpression of TAK1 decreased the expression and phosphorylation of downstream targets such as p38, NLK, and JNK (Hunter et al., 2019). In Dai et al. (2023), we expanded upon this by showing that both overexpression of TAK1 and inhibition of TAK1 phosphorylation, have the same effect on the differentiation of cardiomyocytes. Here, we show that forced repression of TAK1 phosphorylation over a prolonged period results in the upregulation of phospho-TAK1 and several of its downstream targets. We also show that while activation of TAK1, either through norepinephrine stimulation or overexpression, initially activates downstream targets, supraphysiological activation, achieved through combined overexpression and norepinephrine stimulation, conversely leads to the inhibition of phosphorylation in these downstream targets (Fig. 1). This highlights a complex feedback mechanism that tightly regulates TAK1 signaling. Research from various groups has revealed that overexpression of TAK1 may suppress the phosphorylation of downstream targets in a context-dependent fashion (Ajibade et al., 2012; Cheung et al., 2003). Therefore, identifying the downstream targets of TAK1 in various models may yield conflicting results.

Among the many roles of TAK1, recent work has highlighted that TAK1 may be a master regulator of cell survival and death (Mihaly et al., 2014). In particular, genetic studies demonstrate that TAK1 expression is required for the survival of many cell types *in vivo*, including hepatocytes (Inokuchi et al., 2010), hematopoietic cells (Tang et al., 2008), and vascular endothelial cells (Naito et al., 2019). This ability of TAK1 to block cell death pathways is almost certainly due to its kinase activity, as early studies demonstrate that overexpression of kinase-dead versions of TAK1 exacerbates cell death activated by BMP2 (Kimura et al., 2000) and Sef (Yang et al., 2004). In general, it is believed that TAK1 is required to inhibit signaling through either the NF-kB or RIPK-1 pathway (Malireddi et al., 2019; Mihaly et al., 2014; Damhofer et al., 2024). Although we did not examine cell death in these studies, one might expect to see increased cell death in the cachexia model (de Castro et al., 2019). Therefore, further investigation of the control of cell death pathways would be in order, especially for the cachexia model presented in these studies.

Our data suggest that varying outcomes from modulation of TAK1 may be due to either differences in timing or level of expression, context-dependent activation of specific targets in different tissues or different impacts on cells depending on their developmental or physiological state.

## MATERIALS AND METHODS

### Animal models

Longissimus muscle samples were collected from wild-type (Suffolk) and Texel sheep under protocols reviewed and approved by the Clemson University Institutional Animal Care and Use Committee (AUP 2014–081). Snap-frozen tibialis anterior muscle was isolated from KPP model cancer cachexia mice and were collected under protocols reviewed and approved by the Medical University of South Carolina Institutional Animal Care and Use Committee. Cachexia was induced, as described in Talbert et al. (2019).

### Cell culture and ray biotech microarray analysis

R1 mESCs were obtained from the American Tissue Culture Collection (ATCC). TAK1/Map3k7-overexpressing mESCs were previously described, including increased phosphorylation of TAK1 (Brown et al., 2017). mESCs were maintained in a standard growth medium with Leukemia inhibitory factor (LIF) and differentiated as embryoid bodies (EBs), as previously described (Brown et al., 2010). Norepinephrine (0.01uM) was added to phosphorylate TAK1 in both WT and TAK1-overexpressing (TA1OE) EBs, and samples were collected at day 4 for analysis of kinase phosphorylation (Raybiotech kinase array RayBio C-1 Series Human and Mouse MAPK pathway phosphorylation array). Each protein was screened twice in accordance with the suggested protocol from Raybiotech. Protein levels were normalized to controls. Error bars represent the standard deviation of two technical replicates. All ES cell lines are regularly screened for mycoplasma contamination and stemness by differentiation, Oct4 expression and alkaline phosphatase activity. TAK1 overexpressing lines are regularly checked for continued TAK1 overexpression by quantitative PCR and expression of a green fluorescent reporter.

C2C12 cells were cultured in growth medium as described in Yaffe and Saxel (1977), Dulbecco's modification of Eagle's Medium (DMEM) with glutamine supplementation, penicillin/streptomycin, and 20% FBS. At 70% confluence, cells were transferred to differentiation medium, DMEM supplemented with 2% horse serum, 1 μM insulin (added each feeding), antibiotics, and antimycotics (pen/strep) (Blau et al., 1985). On day 2 of differentiation, TAK1 signaling was blocked in experimental samples using 100 nM OXO in dimethyl sulfoxide (DMSO, Sigma-Aldrich) or DMSO alone. Experiments were performed in triplicate. C2C12 cells were a gift from Denis Gutteridge and are tested regularly for retained ability to differentiate into muscle.

### Bradford assay and western blots

Western blots were carried out on Longissimus muscle tissues (LM) analyzed from WT (56-1 SLM, 55-2 LM, 2-1 LM) and Texel sheep (GS1L1 LM, GS8 LM, G19L1 LM), WT and cancer cachexia mice (Talbert et al., 2019) and C2C12 cells with or without the blockade of TAK1 by OXO. Protein was extracted in a radioimmunoprecipitation assay (RIPA) buffer with protease inhibitors and sodium orthovanadate with okadaic acid (10 nM) to preserve phospho-proteins. Total protein was quantified by Bradford assay (Pierce Coomassie Protein Assay Kit) compared to a serial dilution of BSA in a range of 0.064-2 mg/ml. For western blots, 20 μg protein was loaded per well into prepared SDS-PAGE gels (Invitrogen NuPAGE 12% Bis-Tris gels). Proteins were transferred to polyvinylidene difluoride (PVDF) membranes and immunoblotted for visualization with Immobilon Western chemiluminescent HRP substrate (Millipore) using a GeneGnome imaging system and Syngene image analysis software. Quantification was based on raw peak volume data. Antibodies against the following proteins were used: Nemo-like kinase (NLK) (Cell Signaling Technology #94350, 1:1000), phospho-NLK (p-NLK) (biorbyt #orb157946 1:1000), c-Jun N-terminal kinase (JNK) (Cell Signaling Technology #9252, 1:1000), phospho-JNK p-SAPK/JNK (Cell Signaling Technology #4671, 1:1000), p90/Ribosomal s6 kinase (p90RSK) (Cell Signaling Technology #9355T, 1:1000), phospho-RSK (p-p90RSK) (Cell Signaling Technology #9341, 1:1000), p38 (Cell Signaling Technology #9212, 1:1000), phospho-p38 (p-p38) (Cell Signaling Technology #9211T, 1:1000), Heat shock protein 27 (HSP27) (NOVUS #NBP1-75477SS, 1:500), phospho-HSP27 (p-HSP27) (Invitrogen #MA5-37394, 1:1000), cAMP response element binding protein (CREB) (Cell Signaling Technology #9197, 1:1000), phospho-CREB (p-CREB) (Cell Signaling Technology #9198, 1:1000), TAK1 (Sigma-Aldrich, #AB1305414, 1:1000), phospho-TAK1 (p-TAK1) (Invitrogen #MA5-15073, 1:10,000, phospho-glycogen synthase kinase 3b (GSK3B) (Cell Signaling Technology #9323T), 1:1000, phospho-mammalian target of rapamycin (p-mTOR) (Cell Signaling Technology #5536T, 1:1000, vinculin (Abcam #ab130007), myogenic factor 5 (MYF5) (R&D Systems), glyceraldehyde 3-phosphate dehydrogenase (GAPDH) [conjugated with horse radish peroxidase (HRP) (Invitrogen, #MA5-31457, 1:5000)]. HRP-conjugated goat anti-rabbit antibody (Jackson ImmunoResearch #111-035-003 1:10,000) was used as the secondary antibody. In Figs 2, 3, 4 and 6, the panels comprise multiple blots run using the same sets of samples. Equal amounts of samples were loaded in each lane based on a Bradford assay. GAPDH is used to confirm equal loading on blots that were run in parallel. In each of these, the GAPDH blot shown in each figure was used for the normalization of all quantifications. Most blots were used twice. Typically, each blot was probed for a phospho-protein and then probed with a second antibody for a protein with very different molecular masses without blot stripping. Statistical significance was determined by a Student's *t*-test on a minimum of three biological replicates with *P*<0.05 considered significant.

### Immunocytochemistry

On days indicated in the text, C2C12 cells attached to slide wells were fixed in 4% paraformaldehyde and then incubated for 20 min in the blocking buffer (PBS+1% serum, 0.1% BSA, 0.1% triton X-100). Samples were incubated in primary antibody for 1 h (MYF5, R&D Systems, 1:100) at room temperature and then washed three times for 5 min in PBS. Secondary antibody was added (1:1000 anti-goat or anti-rabbit conjugated to Alexa-555 fluorescent protein). Following three rounds of rinses in PBS, the slides were mounted in antifade mounting medium (KPL).

### Statistical methods

Measurements from images were performed using ImageJ and were carried out on a minimum of three independent experiments. Statistical analyses of western blots and metadata collected from images were performed using Microsoft Excel or GraphPad Prism to calculate means and standard deviations. The results are presented as the mean±standard deviation values. For comparisons between the two groups, data were first analyzed for normality using the following statistical tests: D'Agostino and Pearson, Anderson–Darling and Shapiro–Wilk. All underlying data sets in this study passed these normality tests and were then analyzed by a Student's *t*-test. A value of *P*<0.05 was considered statistically significant. *P*<0.05 is indicated by an asterisk (*), *P*<0.01 is indicated by **, *P*<0.001 is indicated by ***, *P*<0.0001 is indicated by ****.

## References

[BIO060487C1] Ajibade, A. A., Wang, Q., Cui, J., Zou, J., Xia, X., Wang, M., Tong, Y., Hui, W., Liu, D., Su, B. et al. (2012). TAK1 negatively regulates NF-κB and p38 MAP kinase activation in Gr-1+CD11b+ neutrophils. *Immunity* 36, 43-54. 10.1016/j.immuni.2011.12.010 22226633 PMC3750978

[BIO060487C2] Biesemann, N., Mendler, L., Kostin, S., Wietelmann, A., Borchardt, T. and Braun, T. (2015). Myostatin induces interstitial fibrosis in the heart via TAK1 and p38. *Cell Tissue Res.* 361, 779-787. 10.1007/s00441-015-2139-2 25725788

[BIO060487C3] Blau, H. M., Pavlath, G. K., Hardeman, E. C., Chiu, C. P., Silberstein, L., Webster, S. G., Miller, S. C. and Webster, C. (1985). Plasticity of the differentiated state. *Science (New York, N.Y.)* 230, 758-766. 10.1126/science.2414846 2414846

[BIO060487C4] Brown, K., Legros, S., Artus, J., Doss, M. X., Khanin, R., Hadjantonakis, A.-K. and Foley, A. (2010). A comparative analysis of extra-embryonic endoderm cell lines. *PLoS One* 5, e12016. 10.1371/journal.pone.0012016 20711519 PMC2919048

[BIO060487C5] Brown, K., Legros, S., Ortega, F. A., Dai, Y., Doss, M. X., Christini, D. J., Robinson, R. B. and Foley, A. C. (2017). Overexpression of Map3k7 activates sinoatrial node-like differentiation in mouse ES-derived cardiomyocytes. *PLoS One* 12, e0189818. 10.1371/journal.pone.0189818 29281682 PMC5744947

[BIO060487C6] Carnac, G., Vernus, B. and Bonnieu, A. (2007). Myostatin in the pathophysiology of skeletal muscle. *Curr. Genomics* 8, 415-422. 10.2174/138920207783591672 19412331 PMC2647158

[BIO060487C7] Chen, M.-M., Zhao, Y.-P., Zhao, Y., Deng, S.-L. and Yu, K. (2021). Regulation of myostatin on the growth and development of skeletal muscle. *Front. Cell Dev. Biol.* 9, 785712. Available at: https://www.frontiersin.org/articles/10.3389/fcell.2021.785712 10.3389/fcell.2021.785712 35004684 PMC8740192

[BIO060487C8] Cheung, P. C. F., Campbell, D. G., Nebreda, A. R. and Cohen, P. (2003). Feedback control of the protein kinase TAK1 by SAPK2a/p38alpha. *EMBO J.* 22, 5793-5805. 10.1093/emboj/cdg552 14592977 PMC275409

[BIO060487C9] Clop, A., Marcq, F., Takeda, H., Pirottin, D., Tordoir, X., Bibé, B., Bouix, J., Caiment, F., Elsen, J.-M., Eychenne, F. et al. (2006). A mutation creating a potential illegitimate microRNA target site in the myostatin gene affects muscularity in sheep. *Nat. Genet.* 38, 813-818. 10.1038/ng1810 16751773

[BIO060487C10] Dai, Y., Nasehi, F., Winchester, C. D. and Foley, A. C. (2023). Tbx5 overexpression in embryoid bodies increases TAK1 expression but does not enhance the differentiation of sinoatrial node cardiomyocytes. *Biol. Open* 12, bio059881. 10.1242/bio.059881 37272627 PMC10261723

[BIO060487C11] Damhofer, H., Tatar, T., Southgate, B., Scarneo, S., Agger, K., Shlyueva, D., Uhrbom, L., Morrison, G. M., Hughes, P. F., Haystead, T. et al. (2024). TAK1 inhibition leads to RIPK1-dependent apoptosis in immune-activated cancers. *Cell Death Dis.* 15, 273. 10.1038/s41419-024-06654-1 38632238 PMC11024179

[BIO060487C12] de Castro, G. S., Simoes, E., Lima, J. D. C. C., Ortiz-Silva, M., Festuccia, W. T., Tokeshi, F., Alcântara, P. S., Otoch, J. P., Coletti, D. and Seelaender, M. (2019). Human cachexia induces changes in mitochondria, autophagy and apoptosis in the skeletal muscle. *Cancers* 11, 1264. 10.3390/cancers11091264 31466311 PMC6770124

[BIO060487C13] Hindi, S. M., Sato, S., Xiong, G., Bohnert, K. R., Gibb, A. A., Gallot, Y. S., McMillan, J. D., Hill, B. G., Uchida, S. and Kumar, A. (2018). TAK1 regulates skeletal muscle mass and mitochondrial function. *JCI Insight* 3, 98441. 10.1172/jci.insight.98441 29415881 PMC5821216

[BIO060487C14] Hunter, A., Dai, Y., Brown, K. J., Muise-Helmericks, R. C. and Foley, A. C. (2019). TAK1/Map3k7 enhances differentiation of cardiogenic endoderm from mouse embryonic stem cells. *J. Mol. Cell. Cardiol.* 137, 132-142. 10.1016/j.yjmcc.2019.10.002 31668971 PMC6905386

[BIO060487C15] Inokuchi, S., Aoyama, T., Miura, K., Österreicher, C. H., Kodama, Y., Miyai, K., Akira, S., Brenner, D. A. and Seki, E. (2010). Disruption of TAK1 in hepatocytes causes hepatic injury, inflammation, fibrosis, and carcinogenesis. *Proc. Natl Acad. Sci. USA* 107, 844-849. 10.1073/pnas.0909781107 20080763 PMC2818947

[BIO060487C16] Kimura, N., Matsuo, R., Shibuya, H., Nakashima, K. and Taga, T. (2000). BMP2-induced apoptosis is mediated by activation of the TAK1-p38 kinase pathway that is negatively regulated by Smad6. *J. Biol. Chem.* 275, 17647-17652. 10.1074/jbc.M908622199 10748100

[BIO060487C17] Liu, N., Williams, A. H., Maxeiner, J. M., Bezprozvannaya, S., Shelton, J. M., Richardson, J. A., Bassel-Duby, R. and Olson, E. N. (2012). microRNA-206 promotes skeletal muscle regeneration and delays progression of Duchenne muscular dystrophy in mice. *J. Clin. Invest.* 122, 2054-2065. 10.1172/JCI62656 22546853 PMC3366415

[BIO060487C18] Malireddi, R. K. S., Kesavardhana, S. and Kanneganti, T.-D. (2019). ZBP1 and TAK1: master regulators of NLRP3 inflammasome/pyroptosis, apoptosis, and necroptosis (PAN-optosis). *Front. Cell Infect. Microbiol.* 9, 406. 10.3389/fcimb.2019.00406 Available at:<https://www.frontiersin.org/journals/cellular-and-infection-microbiology/articles/10.3389/fcimb.2019.00406>. 31850239 PMC6902032

[BIO060487C19] Mihaly, S. R., Ninomiya-Tsuji, J. and Morioka, S. (2014). TAK1 control of cell death. *Cell Death Differ.* 21, 1667-1676. 10.1038/cdd.2014.123 25146924 PMC4211365

[BIO060487C20] Naito, H., Iba, T., Wakabayashi, T., Tai-Nagara, I., Suehiro, J.-I., Jia, W., Eino, D., Sakimoto, S., Muramatsu, F., Kidoya, H. et al. (2019). TAK1 prevents endothelial apoptosis and maintains vascular integrity. *Dev. Cell* 48, 151-166.e7. 10.1016/j.devcel.2018.12.002 30639056

[BIO060487C21] Roy, A., Koike, T. E., Joshi, A. S., Tomaz da Silva, M., Mathukumalli, K., Wu, M. and Kumar, A. (2023). Targeted regulation of TAK1 counteracts dystrophinopathy in a DMD mouse model. *JCI Insight* 8, e164768. 10.1172/jci.insight.164768 37071470 PMC10322678

[BIO060487C22] Srivastava, A. K., Qin, X., Wedhas, N., Arnush, M., Linkhart, T. A., Chadwick, R. B. and Kumar, A. (2007). Tumor necrosis factor-alpha augments matrix metalloproteinase-9 production in skeletal muscle cells through the activation of transforming growth factor-beta-activated kinase 1 (TAK1)-dependent signaling pathway. *J. Biol. Chem.* 282, 35113-35124. 10.1074/jbc.M705329200 17897957 PMC4154379

[BIO060487C23] Talbert, E. E., Cuitiño, M. C., Ladner, K. J., Rajasekerea, P. V., Siebert, M., Shakya, R., Leone, G. W., Ostrowski, M. C., Paleo, B., Weisleder, N. et al. (2019). Modeling human cancer-induced cachexia. *Cell Rep.* 28, 1612-1622.e4. 10.1016/j.celrep.2019.07.016 31390573 PMC6733019

[BIO060487C24] Talebi, R., Ghaffari, M. R., Zeinalabedini, M., Abdoli, R. and Mardi, M. (2022). Genetic basis of muscle-related traits in sheep: a review. *Anim. Genet.* 53, 723-739. 10.1111/age.13266 36184760

[BIO060487C25] Tang, M., Wei, X., Guo, Y., Breslin, P., Zhang, S., Zhang, S., Wei, W., Xia, Z., Diaz, M., Akira, S. et al. (2008). TAK1 is required for the survival of hematopoietic cells and hepatocytes in mice. *J. Exp. Med.* 205, 1611-1619. 10.1084/jem.20080297 18573910 PMC2442639

[BIO060487C26] Yaffe, D. and Saxel, O. (1977). Serial passaging and differentiation of myogenic cells isolated from dystrophic mouse muscle. *Nature* 270, 725-727. 10.1038/270725a0 563524

[BIO060487C27] Yang, X., Kovalenko, D., Nadeau, R. J., Harkins, L. K., Mitchell, J., Zubanova, O., Chen, P.-Y. and Friesel, R. (2004). Sef interacts with TAK1 and mediates JNK activation and apoptosis. *J. Biol. Chem.* 279, 38099-38102. 10.1074/jbc.C400318200 15277532

